# Evaluation and Improvement of Bottlenecking in a Multidisciplinary Oncology Clinic: An Electronic Medical Record Intervention

**DOI:** 10.7759/cureus.4583

**Published:** 2019-05-02

**Authors:** Matthew S Ning, Mary K Dean, Kyle A Taylor, Isidora Arzu, Nicole D Fleming, Neelesh Mutyala, Prakul Suresh, Mark A Lewis, Janet Tu, Victor J Hassid, Timisha Joe, Caitlin Byler, Elizabeth S Bloom, Shalin Shah

**Affiliations:** 1 Radiation Oncology, The University of Texas MD Anderson Cancer Center, Houston, USA; 2 Radiation Oncology, University of Miami, Miami, USA; 3 Miscellaneous, The University of Texas MD Anderson Cancer Center, Houston, USA; 4 Oncology, Intermountain Healthcare, Murray, USA; 5 Gastrointestinal Oncology, The University of Texas MD Anderson Cancer Center, Houston, USA; 6 Plastic Surgery, The University of Texas MD Anderson Cancer Center, Houston, USA

**Keywords:** process optimization, workflow design, outpatient

## Abstract

Purpose: Clinic members reported slower patient flow in the mornings at a multidisciplinary oncology clinic. This study identified the causes of clinic bottlenecking via analysis of patient schedules and transit times, then corrected discrepancies through a quality improvement program.

Methods: Transit times were measured using tracking cards handed out at check-in, marked by each clinic member throughout the encounter, and collected upon discharge. Data were analyzed for differences between morning and afternoon patients, and a Pareto chart was formulated to assess for discrepancies in distribution. Repeat plan-do-study-act (PDSA) cycles were conducted, implementing two changes to redistribute appointments to optimize clinic workflow.

Results: A total of 2951 patient appointments were analyzed: 589 at baseline, 277 following an initial intervention, and 2085 following a subsequent intervention. Analysis of patient transit times revealed no significant differences between morning and afternoon patient groups (t-test, p=.13-.99), with no transit interval markedly longer than others (t-test, p=.32-.83). However, upon evaluation of appointment times, a maldistribution was noted with 57% of patients concentrated between 9:00 am to 12:00 pm, accounting for the perception of bottlenecking. An initial intervention offering patients afternoon appointments on a voluntary basis was insufficient for rebalancing distribution (chi-square test, p=.299); however, an electronic medical record (EMR) intervention with rigid appointment templates was successful (chi-square test, p<.001).

Conclusion: An imbalance of appointment times contributed to the perception of slow clinic throughput. This study emphasizes the importance of systematically investigating even consensus observations for validity prior to costly interventions. Furthermore, these results support the utility of information technology in optimizing clinic workflow.

## Introduction

Extensive waiting times and condensed doctor appointments are associated with low patient satisfaction [1]. Furthermore, workflow inefficiency leads to high job strain and occupational stress for clinic staff, correlating with depression and burnout [2-3]. At our multidisciplinary oncology clinic, multiple clinic team members consistently reported slower clinic throughput during the morning hours. Recurring concerns led to the suggestion of hiring additional personnel to mitigate this bottlenecking within the clinic.

Within oncology, medicine, and life in general, consensus perceptions may dissipate under rigorous scientific evaluation [4]. Prior to implementing a potentially costly intervention, we objectively assessed clinic transit times for bottlenecking and inefficiency throughout our clinic to validate these concerns. Areas of concern were subsequently addressed through feasible quality improvement (QI) initiatives.

## Materials and methods

After QI Board approval, we prospectively followed patients at the University of Texas MD Anderson Cancer Center (MDACC) in Sugar Land during standard outpatient follow-up visits over a two-week period in February 2014 by the following services: medical oncology, breast surgery, gynecologic surgery, colorectal surgery, and genitourinary surgery. The multidisciplinary project team included front desk personnel, lab technicians, business access teams, nursing assistants, clinical nurses, mid-level providers (APPs), and physicians. Patients arriving for new consultations, chemotherapy infusions, or radiation oncology appointments were excluded from the transit time analysis.

Patient transit times were measured via tracking of “pink” cards, handed to patients upon check-in by front desk personnel, timestamped by each team member throughout the encounter, and collected upon discharge. Recorded transit times encompassed check-in through discharge (DC), including the intervals between check-in and vitals, vitals and nurse visit, nurse and APP/physician visit, APP and MD visits (if applicable), and provider and DC. The time interval between check-in and labs was recorded as well (if applicable). The discrepancy between scheduled appointment time and check-in was also collected.

Data were entered into a protected, de-identified database and divided into morning (8:00 am - 11:59 am) and afternoon (12:00 pm - 4:00 pm) appointment groups. Unequal variance t-tests were employed to assess for differences between transit times among groups. Appointment times were plotted on a Pareto chart to further evaluate scheduling distribution. Early appointments (6:00 am - 8:00 am) were excluded from analysis; and while 4:00 pm to 5:00 pm was technically available for appointments, this time slot was rarely utilized at the time of initial data collection.

Following data analysis, a plan-do-study-act (PDSA) cycle was initiated to redistribute appointment times. The initial intervention entailed offering follow-up, chemotherapy consent, and laboratory visits as afternoon appointments on a voluntary basis. To promote this initiative, clinic team members wore buttons advertising “Ask Me How You Can Save Time,” encouraging patients to inquire about scheduling flexibility. Following three months of implementation, measurements and analyses were repeated.

After our institution upgraded its electronic medical record (EMR), a subsequent PDSA cycle was employed to further optimize appointment scheduling. This initiative entailed utilization of the Cadence scheduling application (Epic Systems Corporation, Madison, Wisconsin) within the EMR system. Through provider preference settings, same-day functionality, open access scheduling, and security features, the Cadence scheduling application facilitated equal distribution of appointment times. In February 2018, measurements and analyses were repeated over an extended period to ensure sustainability. Following each intervention, the distribution of appointments (by one-hour intervals from 8:00 am - 5:00 am) was compared to that expected from the baseline measurement via Chi-Square goodness of fit test.

## Results

**Figure 1:**
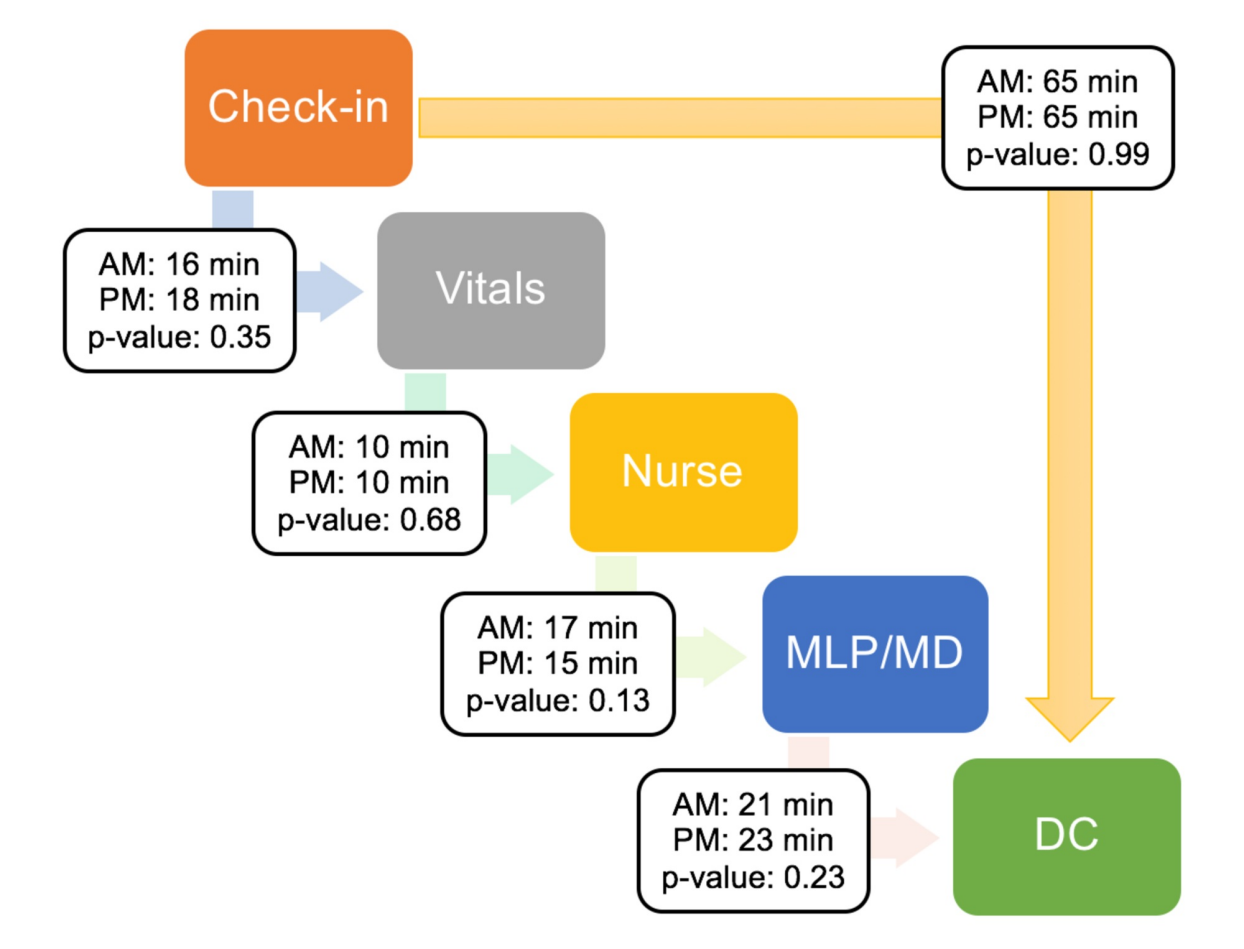
Mean transit times for morning (AM) and afternoon (PM) groups with results of t-test comparisons MLP: mid-level provider; MD: physician; DC: discharge; min: minutes.

A total of 2951 patients were analyzed: 589 at baseline measurements, 277 following initial intervention, and 2085 following final intervention. For the transit time measurements, 243 patients were assessed; and after exclusions, 211 patients were included in the data analysis. All patients either saw an APP and/or physician during the visit. There were no statistically significant differences in any of the clinic transit times between the two groups (morning vs. afternoon), including time differences between: check-in and vitals (mean difference (MD): -2 min; 95% confidence interval (CI): -6 to 2 min; p-value: 0.35), vitals and nurse visit (MD: 0.5 min; 95% CI: -2 to 3 min; p-value: 0.68), nurse and APP/physician visit (MD: 3 min; 95% CI: -1 to 6 min; p-value: 0.13), provider and DC (MD: -2 min; 95% CI: -6 to 1 min; p-value: 0.23), and check-in and DC overall (MD: 0 min; 95% CI: -8 to 8 min; p-value: 0.99). Figure 1 displays these mean clinic transit results with t-test findings.

Furthermore, there was not one specific transit interval markedly longer than the others to account for an area of “bottlenecking”. The average interval between: scheduled appointment time and actual check-in was -9 min for the morning (AM) group (range: -90 to 87 min; median: -10 min; standard deviation (SD): ± 29 min), versus -10 min for the afternoon (PM) group (range: -197 to 137 min; median: -6 min; SD: ± 38 min), and thus not significantly different (MD: 1 min; 95% CI: -9 to 12 min; p=0.83). The average difference between check-in and labs was -9 min for the AM group (range: -71 to 27 min; median: -7 min; SD: ± 11 min), versus -16 min for the PM group (range: -144 to -3 min; median: -7 min; SD: ± 32 min), again non-significant via t-test (MD: 7 min; 95% CI: -7 to 21 min; p=0.32). However, a total of 52% of patients in the AM group had lab work performed, versus only 35% in the PM group.

**Figure 2:**
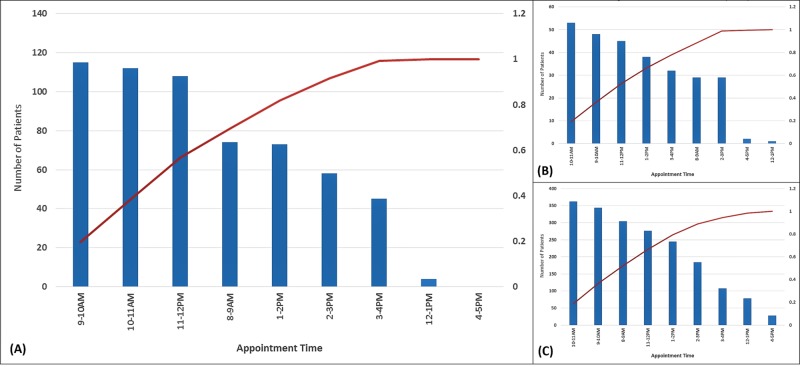
Pareto chart A) initial baseline patients grouped by appointment intervals (n=589); B) following first intervention (n=277); and C) following electronic medical record (EMR) intervention (n=1929). Distribution of late morning (9:00 am to 12:00 pm) appointments decreased from 57% to 51% following the EMR intervention through improved even distribution among all available time slots, thereby balancing clinic load (p<.001) *Chi-Square goodness of fit test.

For the appointment distribution analysis, 277 patients were evaluated following the first intervention, and 1929 after the second intervention (following exclusions). Figure 2A shows the Pareto chart of appointment times grouped in one-hour intervals from the initial data collection (n=589), while Figure 2B and Figure 2C demonstrate the Pareto charts following the initial (n=277) and subsequent (n=1929) interventions, respectively. The baseline distribution identified a large proportion of appointments (57%) allocated to the 9:00 am to 12:00 pm timeslots. The distribution of appointments following the initial intervention was not significantly different than that expected from the baseline distribution, with 53% of appointments scheduled between 9:00 am to 12:00 pm (p=.299); however, the distribution following the EMR intervention did reach significance, with further decrease to 51% of appointments between those times through increased utilization of all available timeslots, thereby evenly distributing clinic load (p<.001).

## Discussion

This study investigated the perception of morning bottlenecking within our multidisciplinary oncology clinic via analysis of patient schedules and transit times, then conducted repeat PDSA cycles to redistribute scheduling, demonstrating that: (1) an imbalance of appointment times contributed to the perception of slow clinic throughput, emphasizing the importance of systematically investigating observations; and (2) information technology (IT) QI initiatives are feasible and can result in sustainable workflow improvements even across a multidisciplinary clinic setting.

Multiple clinic team members raised concerns about the perceived bottlenecking of clinic throughput in the mornings, almost resulting in the hiring of additional staff to mitigate this “problem.” However, our study demonstrated no significant differences in transit times between the morning and afternoon patient groups. Furthermore, none of the transit times was markedly longer than the others, including the time interval suggested to be most problematic by staff: check-in to vital signs. Overall, this study emphasizes the importance of properly investigating even consensus observations prior to implementing costly clinic changes.

While no differences were found with respect to the transit times, the Pareto chart revealed an asymmetric distribution of appointment times, with 57% of appointments scheduled between 9:00 am to noon. Thus, we attributed the bottlenecking perception to this imbalance [5] and sought to redistribute appointment times via QI initiatives to improve the clinic workplace environment for our staff members.

The “Ask Me How You Can Save Time” intervention was an attempt to redistribute appointments on a patient voluntary basis. On inspection of the post-intervention Pareto chart, some trend towards improvement was noted, though this change was non-significant. Following repeat measurement and analyses, our workgroup concluded that relying upon patient volunteerism alone is likely inadequate for resolution of the scheduling maldistribution. Furthermore, over the ensuing months, concerns regarding the sustainability of the intervention arose, particularly regarding the staff efforts required to continuously promote the initiative and manually reschedule follow-up appointments.

After MDACC Sugarland implemented the Epic EMR system, we instead sought a feasible, sustainable IT intervention to further optimize clinic workflow. The scheduling application, Cadence, allows for provider preferences, has same day functionality for open-access scheduling, and hosts security features that can prevent overbooking of time slots [6]. Primary care clinics commonly use open-access scheduling to avoid overbooking [7-8], and other studies have reported improved scheduling with the use of a web-based visual calendar instead of text-based scheduling [9]. Consistent with these data, our utilization of Cadence optimally redistributed appointment times (even in the absence of the previous voluntary initiative), making greater use of previously unpopular slots during which clinic staffing is still available (e.g., 8:00 am - 9:00 am, 12:00 pm - 1:00 pm, and 4:00 pm - 5:00 pm). The sustainability of this IT initiative is also reflected by the extensive data collection of significantly more patients over a longer time interval and at a later time following implementation.

## Conclusions

In summary, this study serves as a proof-of-concept of the feasibility of QI in the setting of a multidisciplinary oncology clinic. While this study took place in a multidisciplinary setting, we believe these results would be reproducible in the setting of a single specialty clinic (e.g., medical oncology) as well. Our findings emphasize the importance of systematically investigating even consensus observations for validity prior to costly interventions. Furthermore, these results support the utility of IT in optimizing clinic workflow and workplace satisfaction.
